# A non-nucleotide agonist that binds covalently to cysteine residues of STING

**DOI:** 10.1247/csf.22085

**Published:** 2022-12-28

**Authors:** Kentaro Matsumoto, Shenwei Ni, Hiroyuki Arai, Takashi Toyama, Yoshiro Saito, Takehiro Suzuki, Naoshi Dohmae, Kojiro Mukai, Tomohiko Taguchi

**Affiliations:** 1 Laboratory of Organelle Pathophysiology, Department of Integrative Life Sciences, Graduate School of Life Sciences, Tohoku University, Sendai, Japan; 2 Department of Health Chemistry, Graduate School of Pharmaceutical Sciences, University of Tokyo, Tokyo, Japan; 3 Laboratory of Molecular Biology and Metabolism, Graduate School of Pharmaceutical Sciences, Tohoku University, Sendai, Japan; 4 Biomolecular Characterization Unit, Technology Platform Division, RIKEN Center for Sustainable Resource Science, Saitama, Japan

**Keywords:** STING agonist, cysteine modification, innate immunity, phenylarsine oxide

## Abstract

Stimulator of interferon genes (STING) is an ER-localized transmembrane protein and the receptor for 2',3'-cyclic guanosine monophosphate–adenosine monophosphate (cGAMP), which is a second messenger produced by cGAMP synthase (cGAS), a cytosolic double-stranded DNA sensor. The cGAS-STING pathway plays a critical role in the innate immune response to infection of a variety of DNA pathogens through the induction of the type I interferons. Pharmacological activation of STING is a promising therapeutic strategy for cancer, thus the development of potent and selective STING agonists has been pursued. Here we report that mouse STING can be activated by phenylarsine oxide (PAO), a membrane permeable trivalent arsenic compound that preferentially reacts with thiol group of cysteine residue (Cys). The activation of STING with PAO does not require cGAS or cGAMP. Mass spectrometric analysis of the peptides generated by trypsin and chymotrypsin digestion of STING identifies several PAO adducts, suggesting that PAO covalently binds to STING. Screening of STING variants with single Cys to serine residues (Ser) reveals that Cys88 and Cys291 are critical to the response to PAO. STING activation with PAO, as with cGAMP, requires the ER-to-Golgi traffic and palmitoylation of STING. Our results identify a non-nucleotide STING agonist that does not target the cGAMP-binding pocket, and demonstrate that Cys of STING can be a novel target for the development of STING agonist.

## Introduction

STING is an ER-localized transmembrane protein essential for control of infections of DNA viruses and tumor immune surveillance (Ishikawa and Barber, 2008; Barber, 2015). After its binding to cGAMP (Wu *et al.*, 2013), which is generated by cGAS in the presence of cytosolic DNA (Sun *et al.*, 2013), STING translocates to the trans-Golgi network (TGN), where STING recruits TBK1 from the cytosol and triggers the type I interferon and proinflammatory responses through the activation of interferon regulatory factor 3 (IRF3) and nuclear factor-kappa B (NF-κB) (Mukai *et al.*, 2021). Activation of TBK1 by STING requires its palmitoylation and the Golgi lipid raft (Mukai *et al.*, 2016; Takahashi *et al.*, 2021), which is composed of cholesterol and sphingomyelin (Simons and Ikonen, 1997). Given that protein palmitoylation has been implicated in the clustering of a number of proteins (Linder and Deschenes, 2007) such as H-ras and Fas into lipid rafts, palmitoylated STING may cluster in the lipid rafts in the TGN, which then facilitates the phosphorylation of TBK1 and IRF3 by bringing them close to each other.

Phosphatidylinositol-4-phosphate (PI4P) is the most abundant phosphoinositide in cells (Balla, 2013). Initially identified as a key phosphoinositide that controls membrane trafficking at the Golgi and the actin cytoskeleton in yeast (Cleves *et al.*, 1989), PI4P soon emerged as a critical regulator of membrane trafficking at the Golgi, also in mammals (Godi *et al.*, 1999). PAO is a membrane permeable trivalent arsenic compound that preferentially reacts with thiol group of Cys and inhibits PI4P synthesis (Wiedemann *et al.*, 1996; Balla *et al.*, 2002). While we performed the experiments to investigate the role of PI4P in the STING trafficking and signalling at the Golgi, we unexpectedly found that PAO could affect STING at the ER and activated the STING signalling pathway.

In the present study, we show that PAO binds covalently to STING at the ER and induces the translocation of STING to the Golgi, where STING activates TBK1 and IRF3 in a STING palmitoylation-dependent manner. Our work identifies a novel STING agonist that does not target the cGAMP-binding pocket, and demonstrates that Cys of STING can be a novel target for the development of STING agonist.

## Results

### PAO activates the STING-TBK1-IRF3 pathway

**Fig. 1:**
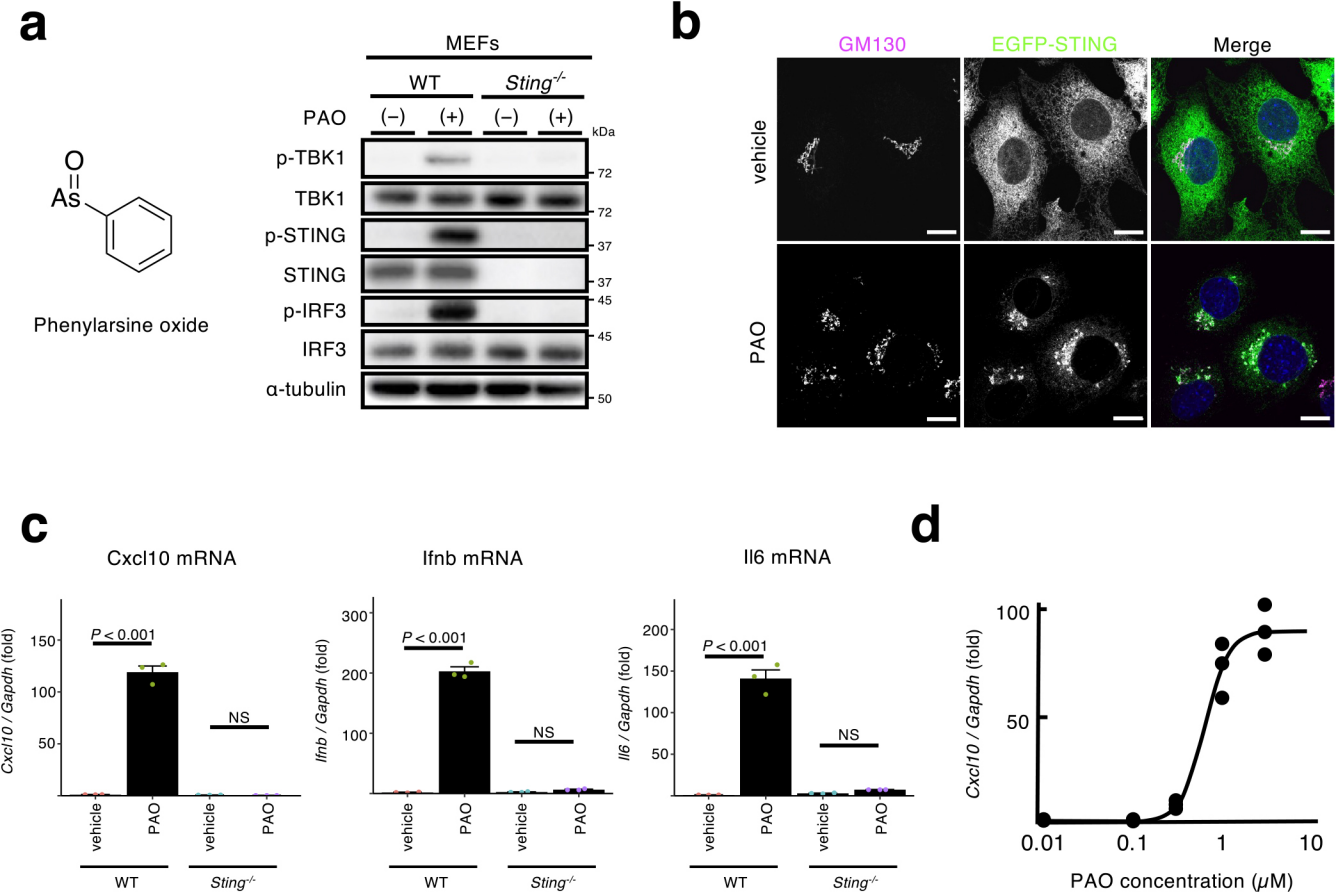
PAO activates the STING pathway (a) MEFs (WT or *Sting*^–/–^) were stimulated with PAO (1 μM) for 1 h. Cell lysates were prepared and analyzed by western blot. (b) Cells were treated with PAO (1 μM) for 1 h, fixed, permeabilized, and stained for GM130 (a Golgi protein, magenta). Nuclei were stained with DAPI (blue). Scale bars, 10 μm. (c) Cells were treated with PAO (1 μM) for 1 h, followed by 4 h incubation without PAO. The expression of *Cxcl10*, *Ifnb*, and *Il6* was quantified by qRT-PCR. (d) Dose-response of *Cxcl10* expression. Data in (c) are mean ± s.e.m. from three independent experiments.

While we performed the experiments to examine the role of PI4P in the STING trafficking and signalling at the Golgi, we happened to find that PAO affected STING at the ER. PAO treatment (1 μM for 1 h) induced the phosphorylation of STING, TBK1, and IRF3 in mouse embryonic fibroblasts (MEFs), but not *Sting^–/–^* (SKO) MEFs (Fig. 1a). To visualize STING, immortalized SKO MEFs were reconstituted with EGFP-tagged mouse STING. As cGAMP (Wu *et al.*, 2013), PAO induced the translocation of STING from the ER to the Golgi (Fig. 1b). Most importantly, PAO induced the expression of the STING-downstream genes, such as *Cxcl10*, *Ifnb*, and *Il6* in a STING-dependent manner (Fig. 1c). PAO exhibited its half-maximum activity at about 1 μM (Fig. 1d). Of note, 1 μM of PAO activated nuclear factor-erythroid 2-related factor 2 (Nrf2) through its Cys modifications (He and Ma, 2009). Given the translocation of STING from the ER to the Golgi by PAO treatment, we reasoned that PAO acted directly on STING, or molecules upstream of STING, such as cGAS/cGAMP.

### cGAS/cGAMP are dispensable for the activation of the STING signalling with PAO

**Fig. 2:**
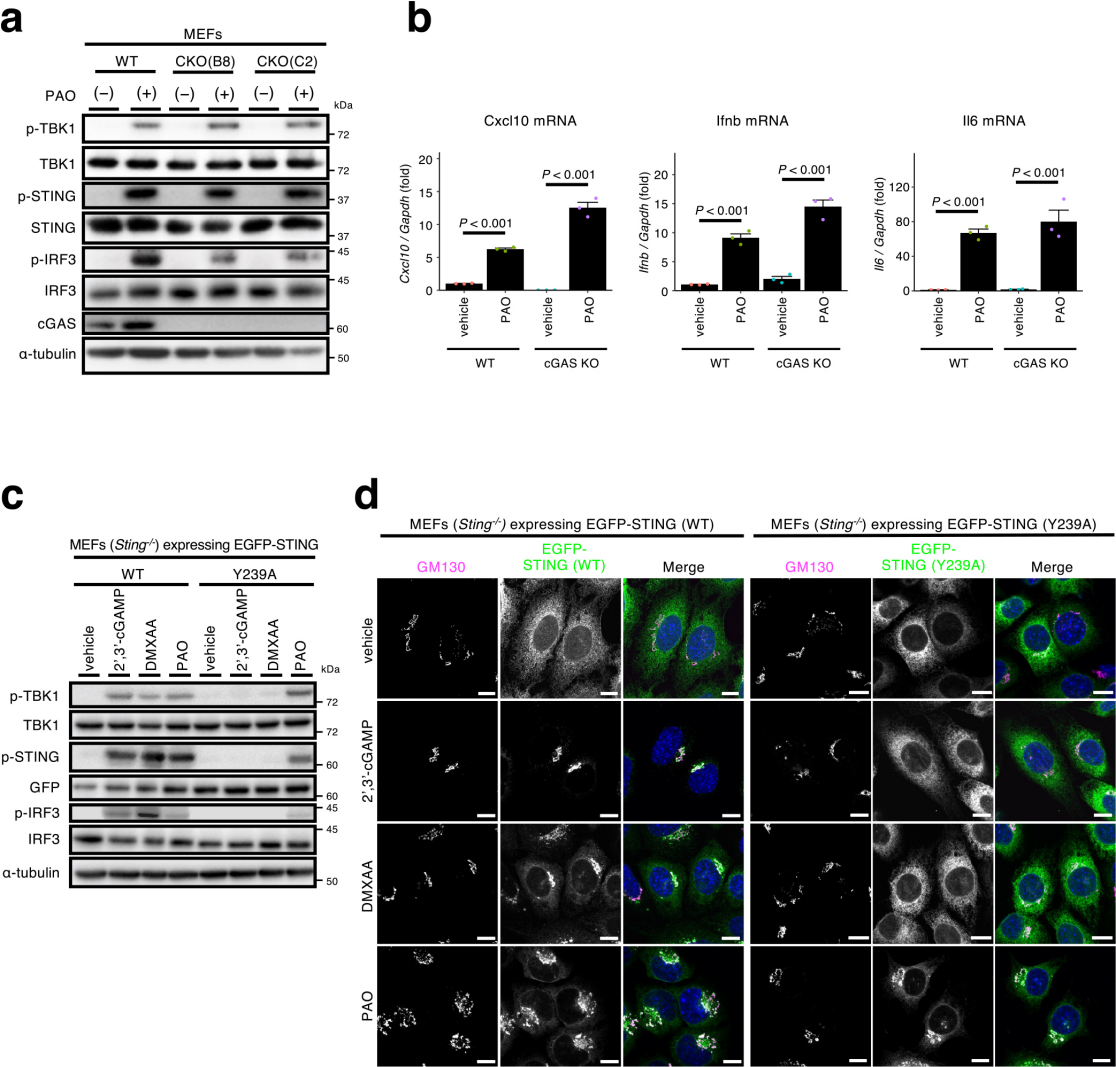
Activation of STING with PAO does not require cGAS/cGAMP (a) cGAS knockout MEFs (clone B8 and clone C2) were stimulated with PAO (1 μM) for 1 h. Cell lysates were prepared and analyzed by western blot. (b) Cells (WT and cGAS KO clone B8) were treated with PAO (1 μM) for 1 h, followed by 4 h incubation without PAO. The expression of *Cxcl10*, *Ifnb*, and *Il6* was quantified by qRT-PCR. (c) *Sting^–/–^* MEFs expressing EGFP-STING (WT or Y239A) were stimulated with 2',3'-cGAMP (2 μg ml^–1^), DMXAA (25 μg ml^–1^), or PAO (1 μM) for 1 h. Cell lysates were prepared and analyzed by western blot. (d) Cells were treated with 2',3'-cGAMP (2 μg ml^–1^), DMXAA (25 μg ml^–1^), or PAO (1 μM) for 1 h, fixed, permeabilized, and stained for GM130 (a Golgi protein, magenta). Nuclei were stained with DAPI (blue). Scale bars, 10 μm . Data in (b) are mean ± s.e.m. from three independent experiments.

PAO may activate cGAS, which produces the STING ligand cGAMP. To examine the role of cGAS in the STING activation with PAO, cGAS-knockout (CKO) MEFs (Mukai *et al.*, 2021) were exploited. As shown (Fig. 2a), PAO induced the phosphorylation of STING, TBK1, and IRF3 in two cell lines (B8 and C2) of CKO MEFs, as wild-type (WT) MEFs. PAO also induced the expression of *Cxcl10*, *Ifnb*, and *Il6* in CKO MEFs (Fig. 2b). These results suggested that cGAS was not required for STING activation with PAO. We further examined the contribution of cGAS/cGAMP using STING (Y239A) variant (Gao *et al.*, 2013), which loses the affinity to cGAMP, and a mouse STING agonist DMXAA. As shown (Fig. 2c), PAO, not cGAMP or DMXAA, induced the phosphorylation of STING, TBK1, and IRF3 in SKO MEFs reconstituted with STING (Y239A). PAO also induced the translocation of STING (Y239A) from the ER to the Golgi (Fig. 2d). These results suggested that activation of the STING pathway with PAO was not via cGAS activation or cGAMP production.

### PAO covalently binds to STING

As PAO makes a covalent bond with Cys of some proteins, PAO may also bind to Cys of STING to exert its effect. Indeed, the treatment of cells with a reducing agent *N*-acetylcysteine (NAC), suppressed PAO-induced phosphorylation of STING, TBK1, and IRF3 (Supplementary Fig. 1), suggesting that PAO activated the STING pathway through cysteine modifications. We particularly focused on Cys88 and Cys91, as these two residues are the specific targets of electrophilic compounds, such as nitro-unsaturated fatty acids (Hansen *et al.*, 2018), H-151 (Haag *et al.*, 2018), and 4-hydroxylnonenal (Jia *et al.*, 2020). We exploited the fact that human STING generated a 8-amino acid peptide (A^87^CLGCPLR^94^) including Cys88 and Cys91 after trypsin digestion, which was successfully identified with mass spectrometry (Hansen *et al.*, 2018; Jia *et al.*, 2020). In contrast to human STING, mouse STING has histidine residue (His) at its position 94, preventing mouse STING from generating the 8-amino acid peptide. Instead, trypsin digestion of mouse STING is expected to yield a longer peptide (A^87^CLGCPIHCMAMILLSSYFYFLQNTADIYLSWMFGLLVLYK^127^), which appeared not suitable for mass spectrometric detection.

We mutated His94 to Arg94 in mouse STING and used the resultant variant (STING H94R) for the following analyses. Mouse STING H94R was confirmed to have the same reactivity as WT STING to PAO, regarding the generation of the phosphorylation of STING, TBK1, and IRF3 (Supplementary Fig. 2a) and the translocation from the ER to the Golgi (Supplementary Fig. 2b).

**Fig. 3:**
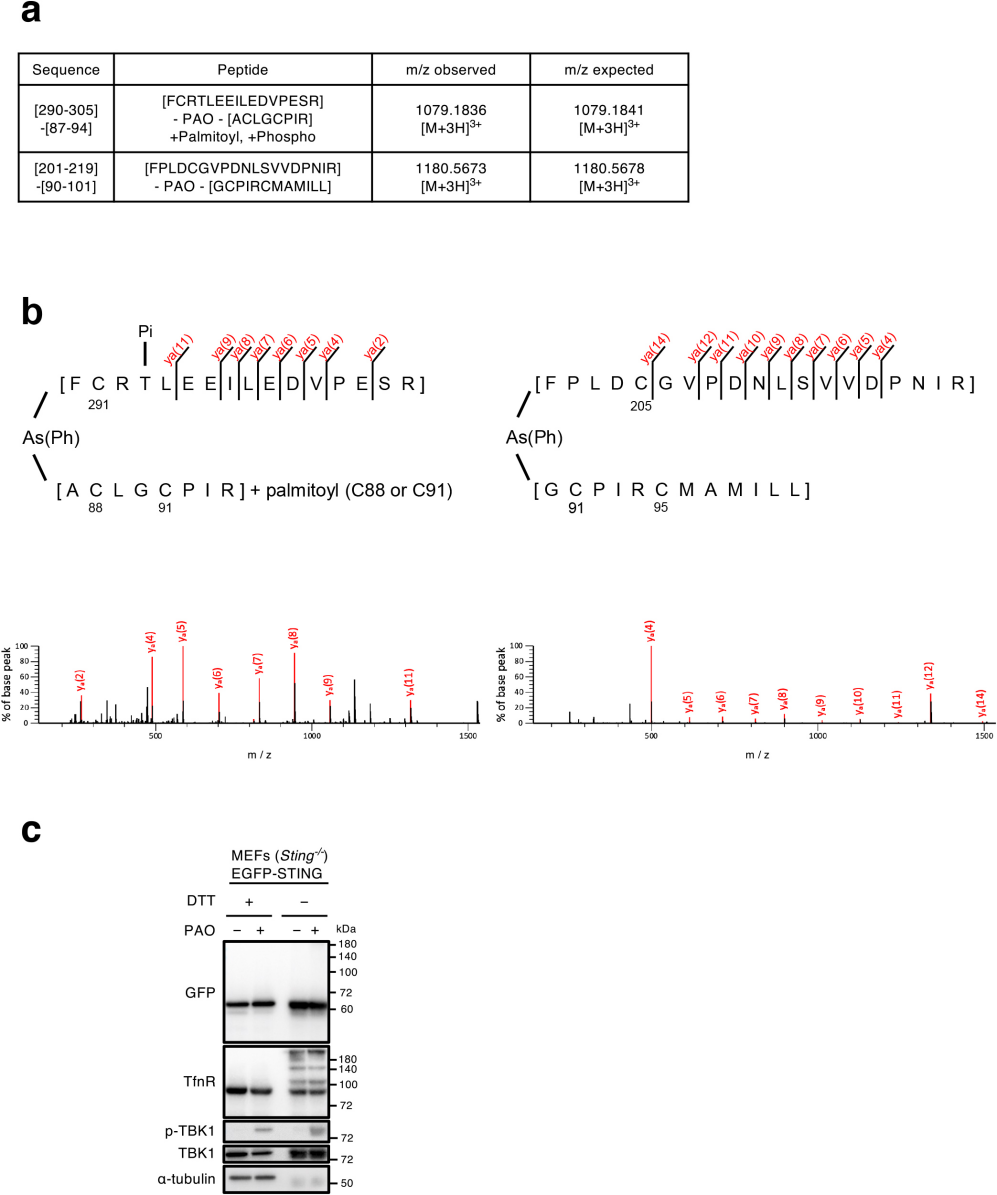
PAO covalently binds to STING (a) *Sting*^–/–^ MEFs expressing EGFP-STING (H94R) were stimulated with PAO (1 μM) for 1 h and lysed. EGFP-STING in the lysates was immunoprecipitated with EGFP nanobody. EGFP-STING were separated with SDS-PAGE under non-reducing conditions and analyzed by mass spectrometry. PAO cross-linked peptides from STING identified by MASCOT cross-linking analysis. The cross-linked peptides consisting of an alpha peptide of 290-FCRTLEEILEDVPESR-305 and a beta peptide of 87-ACLGCPIR-94, an alpha peptide of 201-FPLDCGVPDNLSVVDPNIR-219, and a beta peptide of 90-GCPIRCMAMILL-101 were identified with MASCOT scores of 56 and 69 and expected value of 7.2e – 06 and 1.7.e – 07, respectively. (b) The MSMS spectra of PAO cross-linked peptides of STING. The peaks shown in red were consistent with theoretical values. The y-series ions of the α-peptide were detected, and the parent ions agreed well with the theoretical mass of the PAO cross-linked peptide. (c) Cells were stimulated with PAO (1 μM) for 1 h. Cell lysates were prepared without dithiothreitol (DTT) and analyzed by western blot.

Cells expressing mouse STING H94R tagged with EGFP were treated with PAO for 1 h and lysed. EGFP-STING was immunoprecipitated with anti-GFP-nanobody and eluted from the beads. EGFP-STING was further purified by SDS-PAGE under non-reducing conditions and digested with trypsin and chymotrypsin *in situ*. The resulting peptides were subjected to liquid chromatography-mass spectrometry (LC-MS) analysis, and two PAO-peptide adducts were identified with a MASCOT score higher than 50 using MASCOT cross-linking analysis (Fig. 3a). One of the peptides was acylated with one palmitoyl group and phosphorylated at threonine residue 293. MS/MS spectra of PAO cross-linked peptides are shown in Fig. 3b. The y-series ions of the alpha peptides of [F^290^CRTLEEILEDVPESR^305^] and [F^201^PLDCGVPDNLSVVDPNIR^219^] were observed in agreement with theoretical fragmentation patterns. Although no fragments of the cross-linking partner (beta peptides of [A^87^CLGCPIR^94^] or [G^90^CPIRCMAMILL^101^] were detected, these parent ions of cross-linked peptides agreed with the theoretical masses, suggesting that PAO covalently bounds cysteine residues of STING to form the cross-linking between peptide chains.

To distinguish the intramolecular or intermolecular crosslink of Cys with PAO, EGFP-STING was analyzed by non-reducing SDS-PAGE. As shown (Fig. 3c: lane 2 and lane 4 from the left), the mobility of EGFP-STING was essentially similar both in reducing (with dithiothreitol (DTT))- and non-reducing (without DTT) conditions. No other bands, indicative of dimer or higher oligomers, were detected after PAO stimulation. In contrast to STING, a fraction of transferrin receptor was upshifted in non-reducing conditions, consistently with the fact that transferrin receptor exists as a dimer by disulfide bond (Jing and Trowbridge, 1987). These results suggested that the peptides crosslinked through PAO (Fig. 3a) were formed intramolecularly, not intermoleculary.

### STING C88S and STING C291S variants are not activated with PAO

**Fig. 4:**
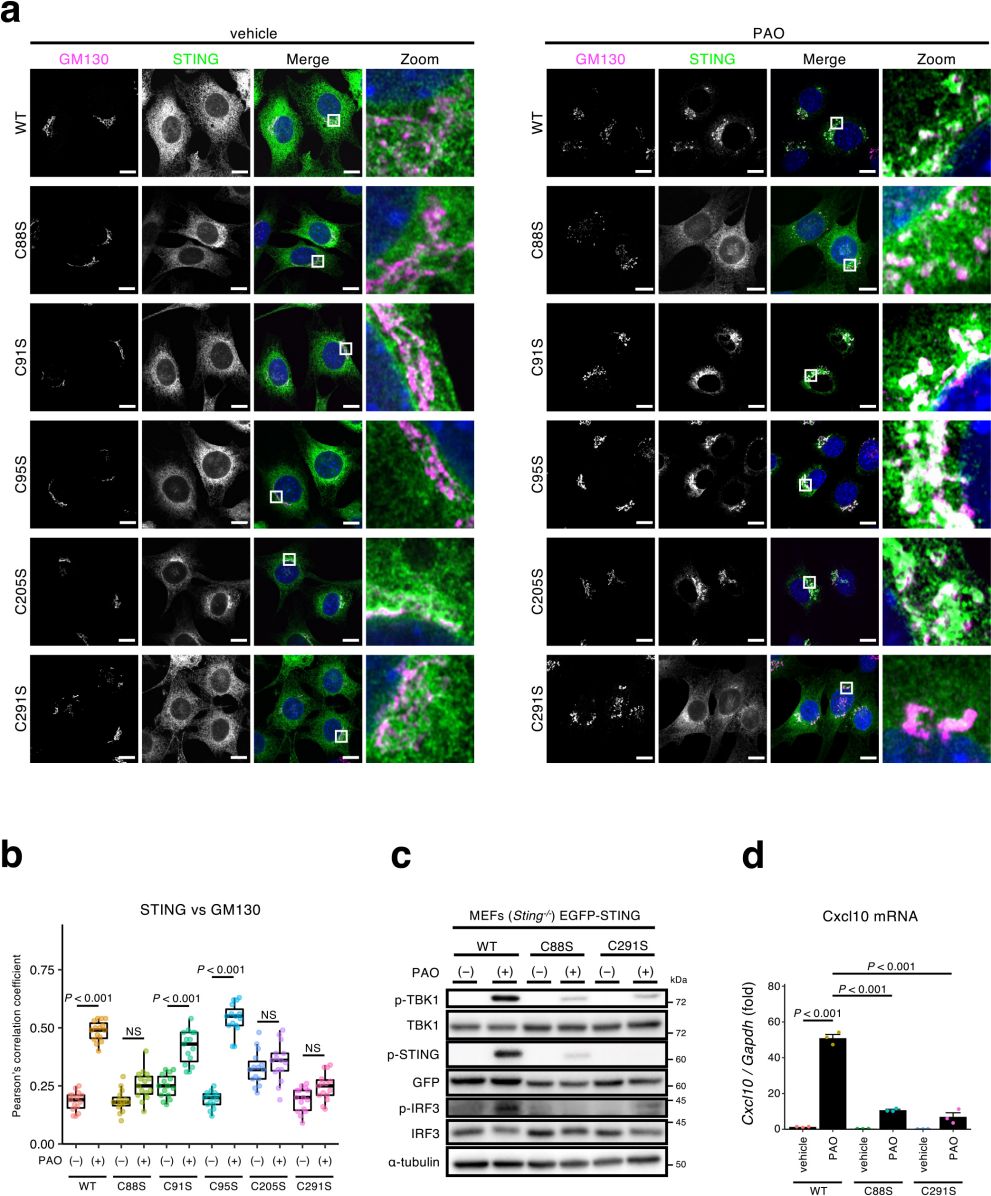
STING variant (C88S or C291S) does not respond to PAO (a) *Sting*^–/–^ MEFs expressing EGFP-STING (C88S, C91S, C95S, C205S, or C291S) were treated with PAO (1 μM) for 1 h. Cells were fixed, permeabilized, and stained for GM130 (a Golgi protein, magenta). Nuclei were stained with DAPI (blue). Scale bars, 10 μm. (b) The Pearson’s correlation coefficient between EGFP-STING and GM130 in (a) is shown. Data are presented in box-and whisker plots (n = 15). (c) *Sting*^–/–^ MEFs expressing EGFP-STING (C88S or C291S) were treated with PAO (1 μM) for 1 h. Cell lysates were then prepared and analyzed by western blot. (d) Cells were treated with PAO (1 μM) for 1 h, followed by 4 h incubation without PAO. The expression of *Cxcl10* was quantified by qRT-PCR. Data are mean ± s.e.m. from three independent experiments.

The mass-spectrometric analysis of the PAO-peptide adducts indicated that Cys205 and Cys291 were covalently modified with PAO. Cys88, Cys91, and/or Cys95 was (were) also indicated to be modified covalently with PAO. We thus examined if which Cys modification(s) with PAO was essential for the STING activation. To address this, we generated STING variants with single Cys to Ser substitution. We found that STING C88S and STING C291S, but not the other variants (STING C91S, STING C95S, and STING C205S) did not translocate to the Golgi (Fig. 4a, b and Supplementary Fig. 3). These results suggested that the modification of Cys88 and Cys291 with PAO was essential for STING activation. STING C88S and STING C291S mostly lacked the activity to phosphorylate TBK1 and IRF3 (Fig. 4c) and to induce the expression of *Cxcl10* (Fig. 4d), further supporting the critical role of Cys88 and Cys291 in activation with PAO. As expected, a STING variant with dual substitution (C88/291S) was also insensitive to PAO stimulation (Supplementary Fig. 4).

Of note, we found that STING C147S and STING C205S appeared to lose the typical ER localization without stimulation and partially accumulated around the perinuclear area, with some co-localization with a Golgi protein GM130 (Fig. 4a, b and Supplementary Fig. 3). Thus, Cys147 and Cys205 may be essential for the steady-state ER localization of STING. In contrast, regarding Cys147, the previous report suggested that Cys148 of human STING (equivalent to mouse Cys147) was essential for the translocation of STING to the Golgi (Ergun *et al.*, 2019). The different behavior of the two Cys147 variants may be due to the species difference and/or the different experimental system used, *i.e.*, MEFs stably expressing STING *vs.* HEK293T cells overexpressing STING.

### STING activation with PAO requires the ER-to-Golgi traffic and palmitoylation

**Fig. 5:**
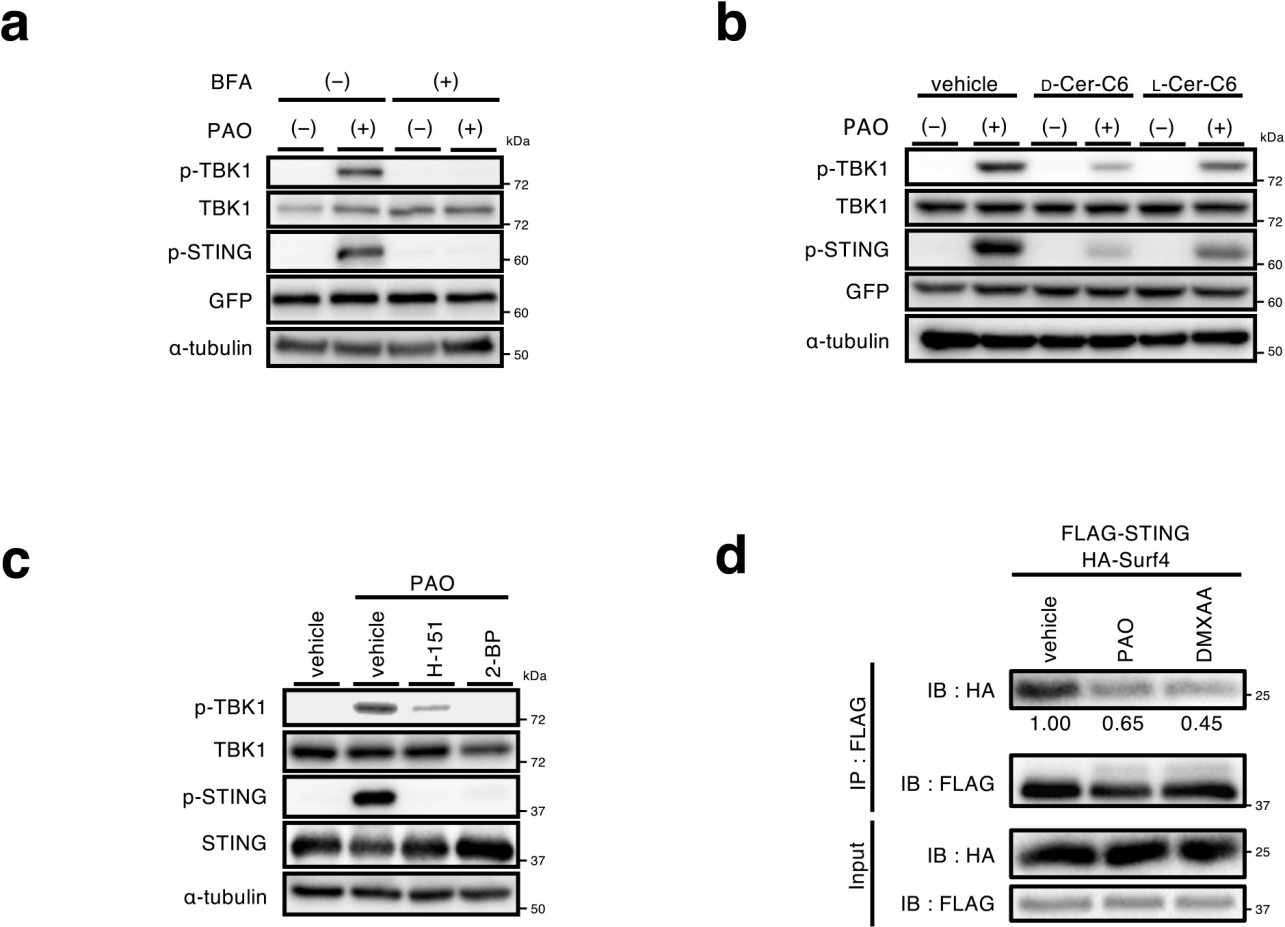
Mechanism underlying activation of STING with PAO (a) *Sting*^–/–^ MEFs expressing EGFP-STING were treated with brefeldin A (BFA) (0.5 μg mL^–1^) for 30 min followed by the stimulation with PAO (1 μM) for 1 h. Cell lysates were then prepared and analyzed by western blot. (b) *Sting*^–/–^ MEFs expressing EGFP-STING were treated with D-C6-ceramide (20 μM) or L-C6-ceramide (20 μM) for 1 h followed by treatment with PAO (1 μM) for 1 h. (c) MEFs were treated with H-151 (10 μM) or 2-bromopalmitate (2-BP) (200 μM) for 3 h followed by the stimulation with PAO (1 μM) for 1 h. (d) HA-Surf4 and FLAG-STING were stably expressed in *Sting*^–/–^ MEFs. Cells were stimulated with PAO (1 μM) or DMXAA (25 μg mL^–1^) for 1 h. Cell lysates were prepared, and FLAG-STING was immunoprecipitated with anti-FLAG antibody. Cell lysates and the immunoprecipitates were analyzed by western blot.

The activation of the STING signalling pathway with cGAMP requires the exocytic membrane traffic from the ER to the Golgi, palmitoylation of STING at the Golgi, and the lipid-raft microdomains at the trans-Golgi network (TGN) (Mukai *et al.*, 2016; Takahashi *et al.*, 2021). The treatment of cells with BFA (a fungal macrocyclic lactone that blocks the ER-to-Golgi traffic) (Lippincott-Schwartz *et al.*, 1990), or D-ceramide-C6 (an inhibitor of the formation of the lipid raft microdomains), suppressed the phosphorylation of STING and TBK1 with PAO (Fig. 5a, b). These results suggested that the activation of the STING signaling pathway with PAO occurred at the Golgi, in particular at the TGN, but not the ER. This notion was further supported by the result that phosphorylation of TBK1, a hallmark of the STING activation, was confined to the TGN (Supplementary Fig. 5). The treatment with H-151 (Haag *et al.*, 2018) (an inhibitor of palmitoylation of STING Cys91) or 2-BP (a pan-palmitoyltransferase inhibitor) also suppressed the phosphorylation of STING and TBK1 with PAO (Fig. 5c). STING C91S had a reduced activity to induce the expression of *Cxcl10* (Supplementary Fig. 6). These results suggested that STING activation by PAO required STING palmitoylation.

Surf4 is a protein that circulates between the ER and the Golgi (Mitrovic *et al.*, 2008). We previously showed that STING was constantly retrieved back from the Golgi to the ER by the binding to Surf4, by which the steady-state localization of STING at the ER was warranted (Mukai *et al.*, 2021). Intriguingly, the amount of Surf4 that bound to STING decreased after treatment with PAO (Fig. 5d). The weaker binding of Surf4 to PAO-conjugated STING may result in inefficient retrograde transport of STING to the ER, leading to the accumulation and activation of STING at the Golgi.

## Discussion

Several studies have demonstrated that STING activity is tightly associated with cellular redox status. Treatment of cells with hydrogen peroxide generated intermolecular disulfide bonds between STING, leading to the formation of inactive STING oligomers (Jin *et al.*, 2010; Tao *et al.*, 2020). Cys64, Cys148, Cys292, and Cys309 of STING were suggested to be involved in the formation of disulfide bonds (Jin *et al.*, 2010). High-resolution proteomics showed that Cys148 was constitutively oxidized, whereas Cys206 was oxidized by hypochlorous acid or by cGAMP (Zamorano Cuervo *et al.*, 2021). Glutathione peroxidase 4 (GPX4) deficiency promotes lipid peroxidation (Ursini *et al.*, 1982). 4-Hydroxynonenal, one of the end products of lipid peroxidation, covalently bound Cys88 of STING and suppressed STING activation by inhibiting palmitoylation of Cys88 (Jia *et al.*, 2020). In the present study, PAO was shown to bind covalently Cys88/Cys291 of STING and to activate STING in the absence of cGAMP. Thus, these studies, including the present one, suggest that cytoplasmic Cys of STING is susceptible to oxidation, which affects STING activity. The molecular basis underlying the specificity of different oxidants to different Cys of STING remains to be elucidated.

Translocation of STING from the ER to the Golgi is essential for the activation of the STING signalling (Ishikawa *et al.*, 2009; Mukai *et al.*, 2016; Taguchi and Mukai, 2019; Kemmoku *et al.*, 2022). There are a number of molecules involved in the translocation process. The exit from the ER requires the coat protein complex-II (COP-II), a protein complex that is responsible for creating membrane vesicles (COP-II vesicles) that bud from the ER (Barlowe and Helenius, 2016). Sar1 (a small GTPase), Sec23/Sec24 (inner coat proteins), and Sec13/Sec31 (outer coat proteins), and several other proteins associated with COP-II vesicles were shown to be involved in the STING translocation (Ogawa *et al.*, 2018; Sun *et al.*, 2018; Ran *et al.*, 2019; Zhang *et al.*, 2020; Gui *et al.*, 2019). The Ca^2+^ sensor stromal interaction molecule 1 (STIM1), an ER-resident protein, was suggested to act as a tether of STING to the ER (Srikanth *et al.*, 2019). Surf4 is a protein that circulates between the ER and the Golgi (Mitrovic *et al.*, 2008). We and others have recently shown that STING was constantly retrieved back from the Golgi to the ER by the binding to Surf4 (Mukai *et al.*, 2021; Deng *et al.*, 2020; Steiner *et al.*, 2022). The PAO binding may induce the conformational change of STING, which strengthens or weaken the affinity of STING to these regulators of the membrane trafficking. Indeed, PAO-conjugated STING had a lower affinity to Surf4 (Fig. 5d).

As mentioned above, we observed PAO-crosslinked peptides, one of which was modified with palmitoyl group (Fig. 3b). Given the critical role of Cys88 in the activation of STING with PAO (Fig. 4c, d), we reasoned that Cys88 was crosslinked to Cys291 with PAO and that the other Cys in the beta peptide [A^87^CLGCPIR^94^], *i.e.*, Cys91, was palmitoylated. The activation of STING with PAO required the ER-to-Golgi traffic, palmitoylation of STING, and the raft-lipid microdomains at the TGN (Fig. 5a–c). These results indicated that PAO-conjugated STING, as cGAMP-bound STING, would cluster in the lipid raft at the TGN with the aid of its palmitoylation, leading to the activation of TBK1 and IRF3 (Mukai *et al.*, 2016).

As shown (Supplementary Fig. 7), PAO could not activate human STING. Human STING also possesses Cys88 and Cys292 (equivalent to mouse Cys291), which can be the targets of PAO. One possibility to account for the inability of PAO to activate human STING is that the three-dimensional distance between Cys88 and Cys292 in human STING may differ from the one between Cys88 and Cys291 in mouse STING. If it is the case, towards the development of human STING agonists with the same mechanism of action as PAO, it will be essential to design the compound that has the appropriate molecular length to crosslink Cys88 and Cys292.

A number of STING agonists have been developed recently (Chin *et al.*, 2022). Most of the STING agonists target the cGAMP-binding pocket, whereas two STING agonists, PC7A (Li *et al.*, 2021) and C53 (Lu *et al.*, 2022), do not. The present study provides another example of STING agonist that does not target the cGAMP-binding pocket, and demonstrates that cytoplasmic Cys of STING can be a novel target for the development of STING agonists, besides STING antagonists (Hansen *et al.*, 2018; Haag *et al.*, 2018; Decout *et al.*, 2021).

## Methods

### Antibodies

Antibodies used in this study were as follows: rabbit anti-phospho-STING (D8F4W, dilution 1:1000), rabbit anti-phospho-TBK1 (D52C2, dilution 1:1000), rabbit anti-IRF3 (D83B9, dilution 1:1000), rabbit anti-phospho-IRF3 (4D4G, dilution 1:1000) (Cell Signaling Technology); rabbit anti-TBK1 (ab40676, dilution 1:1000) (Abcam); mouse anti-α-tubulin (DM1A, dilution 1:1000) (Sigma-Aldrich); Goat Anti-Rabbit IgG (H + L) Mouse/Human ads-HRP (4050-05, dilution 1:10,000) and Goat Anti-Mouse IgG (H + L) Human ads-HRP (1031-05, dilution 1:10,000) (Southern Biotech); sheep anti-TGN38 (AHP499G, dilution 1:200) (Bio-Rad); mouse anti-GM130 (610823, dilution 1:2000) (BD Biosciences).

### Reagents

The following reagents were purchased from the manufacturers as noted: DMXAA (14617, Cayman); nocodazole (13857, Cayman); 2-bromopalmitate (320-76562, Wako); 2',3'-cGAMP (InvivoGen); D-ceramide-C6 (62525, Cayman), L-ceramide-C6 (24388, Cayman); H-151 was provided by Carna Biosciences, Inc.

### Cell culture

MEFs were obtained from embryos of WT or *Sting*^–/–^ mice at E13.5 and immortalized with SV40 Large T antigen. MEFs were cultured in DMEM supplemented with 10% fetal bovine serum (FBS) and penicillin/streptomycin/glutamine (PSG) in a 5% CO_2_ incubator. MEFs that stably express tagged proteins were established using retrovirus. Plat-E cells were transfected with pMXs vectors, and the medium that contains the retrovirus was collected. MEFs were incubated with the medium and then selected with puromycin (2 μg mL^–1^).

### PCR cloning

N-terminal EGFP- or FLAG-tagged mouse STING (NM_028261) was introduced into pMXs-IPuro. STING variants were generated by site-directed mutagenesis.

### Immunocytochemistry

Cells were fixed with 4% paraformaldehyde (PFA) in PBS at room temperature for 15 min, permeabilized with 0.1% Triton X-100 in PBS at room temperature for 5 min. After blocking with 3% BSA in PBS, cells were incubated with primary antibodies. After washing with PBS three times, cells were then incubated with the secondary antibody at room temperature for 60 min, washed, and mounted with ProLong™ Glass Antifade Mountant (P36982, Thermo Fisher Scientific).

### qRT-PCR

Total RNA was extracted from cells using Isogen II (Nippongene) or Superprep II (TOYOBO), and reverse-transcribed using ReverTraAce qPCR RT Master Mix with gDNA Remover (TOYOBO). Quantitative real-time PCR (qRT-PCR) was performed using KOD SYBR qPCR (TOYOBO) and LightCycler 96 (Roche). The sequences of the primers were as follows; 5'-CAGCTCCAAGAAAGGACGAAC-3' (mouse *Ifnb*; sense primer) and 5'-GGCAGTGTAACTCTTCTGCAT-3' (mouse *Ifnb*; antisense primer); 5'-AGTGCTGCCGTCATTTTCTGCCTC-3' (mouse *Cxcl10*; sense primer) and 5'-GCAGGATAGGCTCGCAGGGATGATT-3' (mouse *Cxcl10*; antisense primer); 5'-TAGTCCTTCCTACCCCAATTTC-3' (mouse *Il6*; sense primer) and 5'-TTGGTCCTTAGCCACTCCTTC-3' (mouse *Il6*; antisense primer); 5'-AGGTCGGTGTGAACGGATTTG-3' (mouse *Gapdh*; sense primer) and 5'-TGTAGACCATGTAGTTGAGGTCA-3' (mouse *Gapdh*; antisense primer). Target gene expression was normalized on the basis of *Gapdh* content.

### Immunoprecipitation

Cells were washed with ice-cold PBS and scraped in immunoprecipitation buffer composed of 50 mM HEPES-NaOH (pH 7.2), 150 mM NaCl, 5 mM EDTA, 1% Triton X-100, protease inhibitor cocktail (25955, dilution 1:100) (Nacalai Tesque) and phosphatase inhibitors (8 mM NaF, 12 mM β-glycerophosphate, 1 mM Na_3_VO_4_, 1.2 mM Na_2_MoO_4_, 5 mM cantharidin, 2 mM imidazole). The cell lysates were centrifuged at 15,000 rpm for 15 min at 4°C, and the resultant supernatants were pre-cleared with Ig-Accept Protein G (Nacalai Tesque) at 4°C for 1 h. The lysates were then incubated for 2 h at 4°C with anti-FLAG M2 affinity Gel. The beads were washed four times with immunoprecipitation wash buffer (50 mM HEPES-NaOH (pH 7.2), 150 mM NaCl, 0.1% Triton X-100) and eluted with 2 × Laemmli sample buffer. The immunoprecipitated proteins were separated with SDS-PAGE and transferred to PVDF membrane, then analyzed by western blot.

### Western blotting

Proteins were separated in polyacrylamide gel and then transferred to polyvinylidene difluoride membranes (Millipore). These membranes were incubated with primary antibodies, followed by secondary antibodies conjugated to peroxidase. The proteins were visualized by enhanced chemiluminescence using Fusion SOLO.7S.EDGE (Vilber-Lourmat).

### Mass spectrometry

Purified GFP nanobodies from bacteria were incubated with GST-Accept resin (09277-56, nacalai) at room temperature for 2 h. Cells were lysed with IP buffer (50 mM HEPES-NaOH (pH 7.2), 150 mM NaCl, 5 mM EDTA, 1% Triton X-100, protease inhibitors, and phosphatase inhibitors). The lysates were centrifugated at 15,000 rpm for 10 min at 4°C, and the resultant supernatants were incubated overnight at 4°C with the resin. The beads were washed six times with immunoprecipitation wash buffer (50 mM HEPES-NaOH (pH 7.2), 150 mM NaCl, 1% Triton X-100), and boiled at 95°C for 5 min with elution buffer (50 mM HEPES-NaOH (pH 7.2), 150 mM NaCl, 5 mM EDTA, 1% Triton X-100). Eluted proteins were applied to SDS-PAGE under non-reducing conditions. The gel was stained with CBB and the band that corresponds to STING was excised. The gel pieces were digested with trypsin (tosyl phenylalanyl chloromethyl ketone treated; Worthington Biochemical Co) and chymotrypsin (Cooper Biomedical, Inc) at 37°C for 12 h. The resulting peptides were separated using an Easy nLC 1000 (Thermo Fisher Scientific) equipped with a nano-ESI spray column (NTCC-360, 0.075 mm internal diameter × 100 mm length, 3 μm, Nikkyo Technos Co) at a flow rate of 300 nl min^–1^. The separation was performed with 0.1% formic acid as solvent A and 100% acetonitrile containing 0.1% formic acid as solvent B, with a linear gradient of 40 min with solvent B rising from 0% to 80%. The separated peptides were analyzed using an online coupled Q Exactive Mass Spectrometer (Thermo Fisher Scientific) using the data-dependent Top 10 method. The acquired data were processed using MASCOT 2.8 (Matrix Science) and Proteome Discoverer 2.4 (Thermo Fisher Scientific). The MASCOT search with crosslinking analysis was conducted as follows: Database, in-house database-containing STING sequence; type of search, MS/MS ion; enzyme, trypchymo (trypsin + chymotrypsin); fixed modification, none; variable modifications, oxidation (M), dehydro (C), phospho (ST), palmitoyl (C) and crosslinking with PAO or disulfide; mass values, monoisotopic; peptide mass tolerance, ±15 ppm; fragment mass tolerance, ±30 mmu; max missed cleavages, 4; instrument type, ESI-TRAP.

### Quantification of imaging data

For quantification of imaging data of multiple cells, individual cells were segmented using ROI. Pearson’s correlation coefficient was then quantified by Coloc 2 in Fiji plugin with ROI data.

### Statistical analyses

Error bars displayed in the bar plots throughout this study represent s.e.m. unless otherwise indicated and were calculated from triplicate samples. In box-and-whisker plots, the box bounds the interquartile range (IQR) divided by the median, and whiskers extend to a maximum of 1.5 × IQR beyond the box. The corresponding data points are overlayed on the plots. Statistical significance was determined with one-way ANOVA followed by Tukey-Kramer *post hoc* test.

### Data availability

The data sets generated and/or analyzed during the present study are available from the corresponding author on reasonable request.

## Author Contributions

K.Matsumoto designed and performed the experiments, analyzed the data, interpreted the results, and wrote the paper; S.N. performed experiments with PAO; H.A. discussed the results; T.Toyama and Y.S. designed the experiments with NAC. T.S. and N.D. performed the proteomics analysis; K.Mukai and T.Taguchi designed the experiments, interpreted the results, and wrote the paper.

## Competing Interests

The authors declare no competing financial interests.
