# Supplementary material for: A non-nucleotide agonist that binds covalently to cysteine residues of STING
Source: Cell Struct Funct. 2022 Dec 28;48(1):59–70. doi: 10.1247/csf.22085 (PMC10721953; doi:10.1247/csf.22085)
Supplement: Supplementary file 1 — Supplementary Figures [file csf_48_22085_1.pdf]

Supplementary Fig. 1

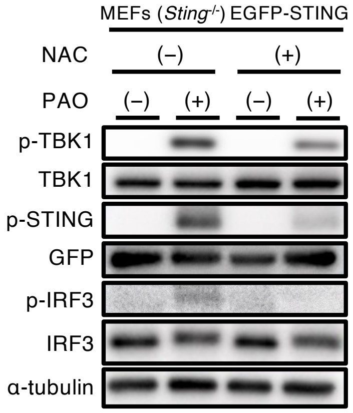

Supplementary Fig. 1 | *N*-acetylcysteine (NAC) suppressed PAO-induced STING activation
Sting$^{-/-}$ MEFs expressing EGFP-STING were treated with *N*-acetyl cysteine (NAC) (2 mM) for 3 h followed by the stimulation with PAO (1 μM) for 1 h. Cell lysates were prepared and analyzed by western blot.

Supplementary Fig. 2

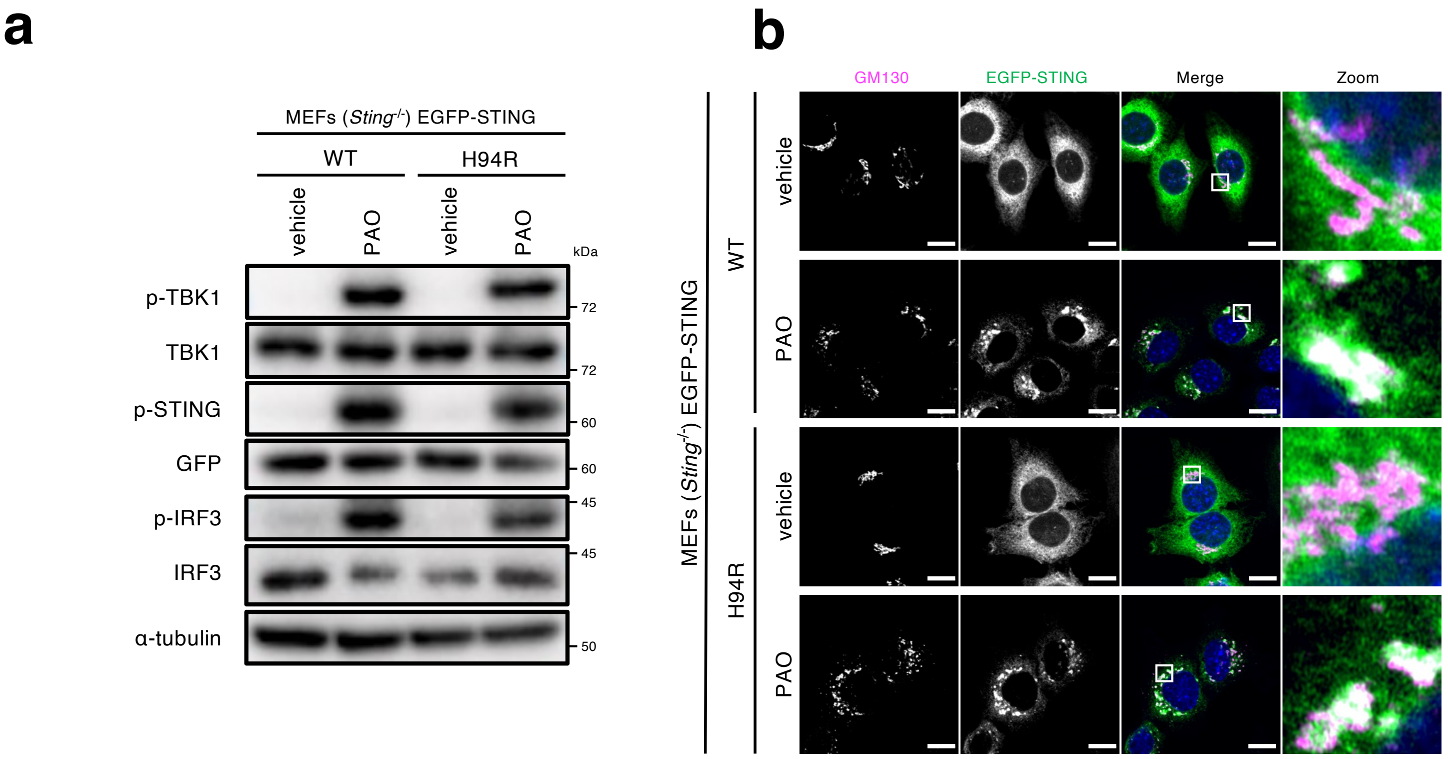

Supplementary Fig. 2 | MEFs expressing EGFP-STING (H94R) had the same reactivity as STING (WT) to PAO
a *Sting*$^{-/-}$ MEFs expressing EGFP-mouse STING (H94R) were treated with PAO (1 μM) for 1 h. Cell lysates were then prepared and analysed by western blot. **b** Cells were treated with PAO (1 μM) for 1 h. Cells were then fixed, permeabilized, and stained for GM130 (a Golgi protein, magenta). Nuclei were stained with DAPI (blue). Scale bars, 10 μm.

Supplementary Fig. 3

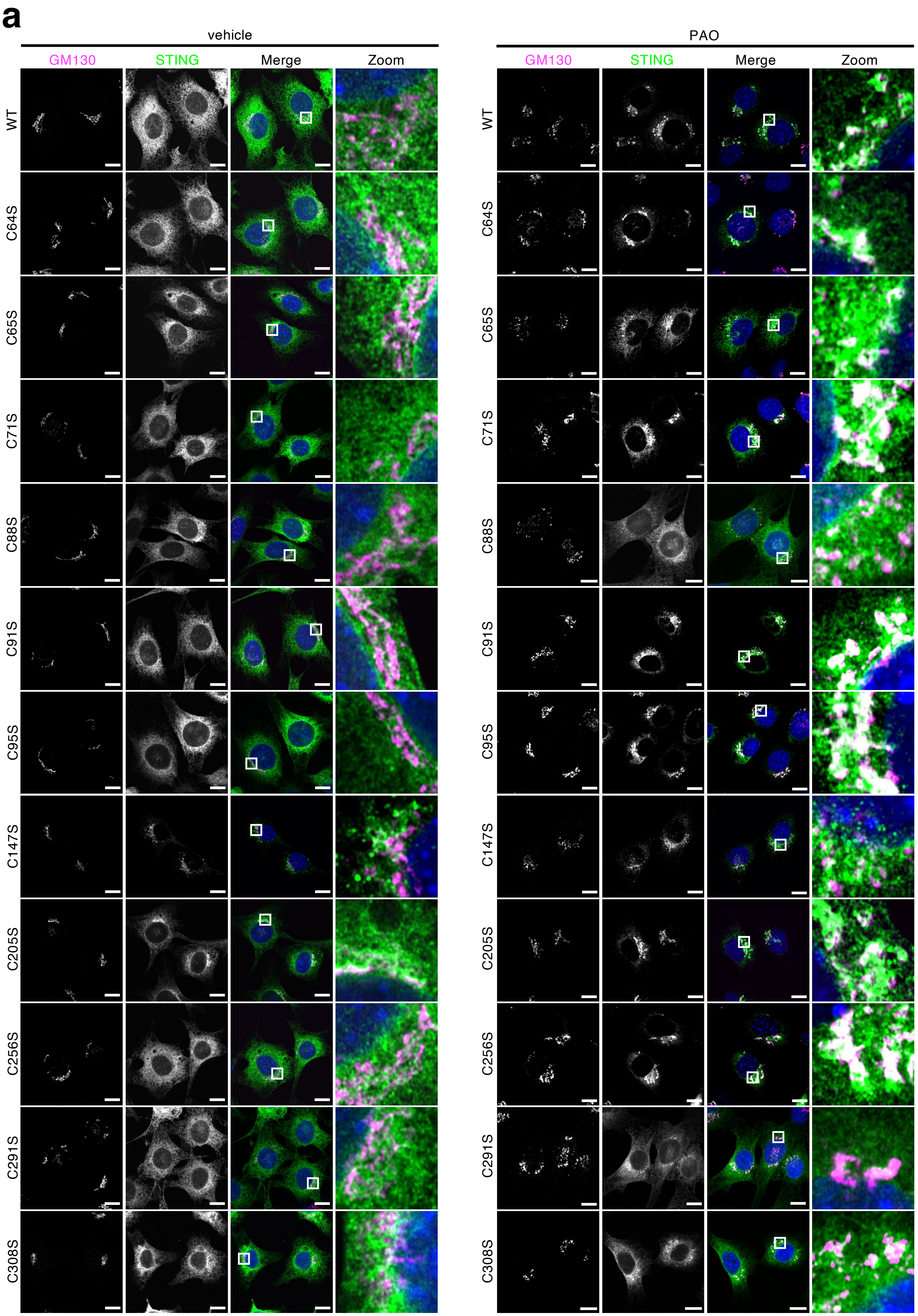

Supplementary Fig. 3 (continued)

b

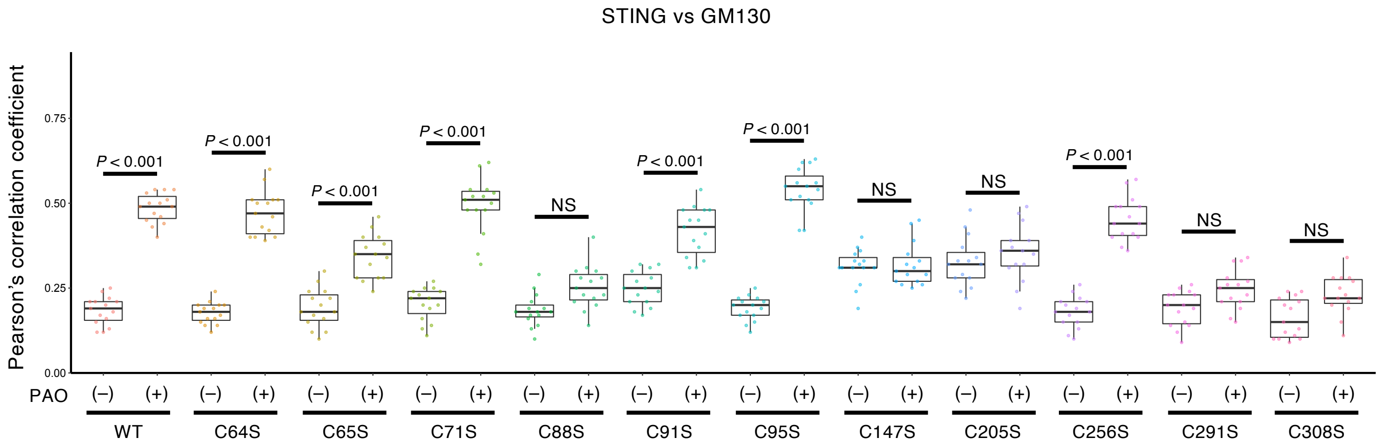

Supplementary Fig. 3 | The effect of Cys to Ser substitutions on translocation of STING by PAO
a EGFP-STING (WT, C64S, C65S, C71S, C88S, C91S, C95S, C147S, C205S, C256S, C291S, C308S) expressing *Sting*$^{-/-}$ MEFs were treated with PAO (1 µM) for 1 h. Cells were fixed, permeabilized, and stained for GM130 (a Golgi protein, magenta). Nuclei were stained with DAPI (blue). Scale bars, 10 µm. **b** The Pearson's correlation coefficient between EGFP-STING and GM130 in (a) is shown. Data are presented in box-and whisker plots (n = 15).

Supplementary Fig. 4

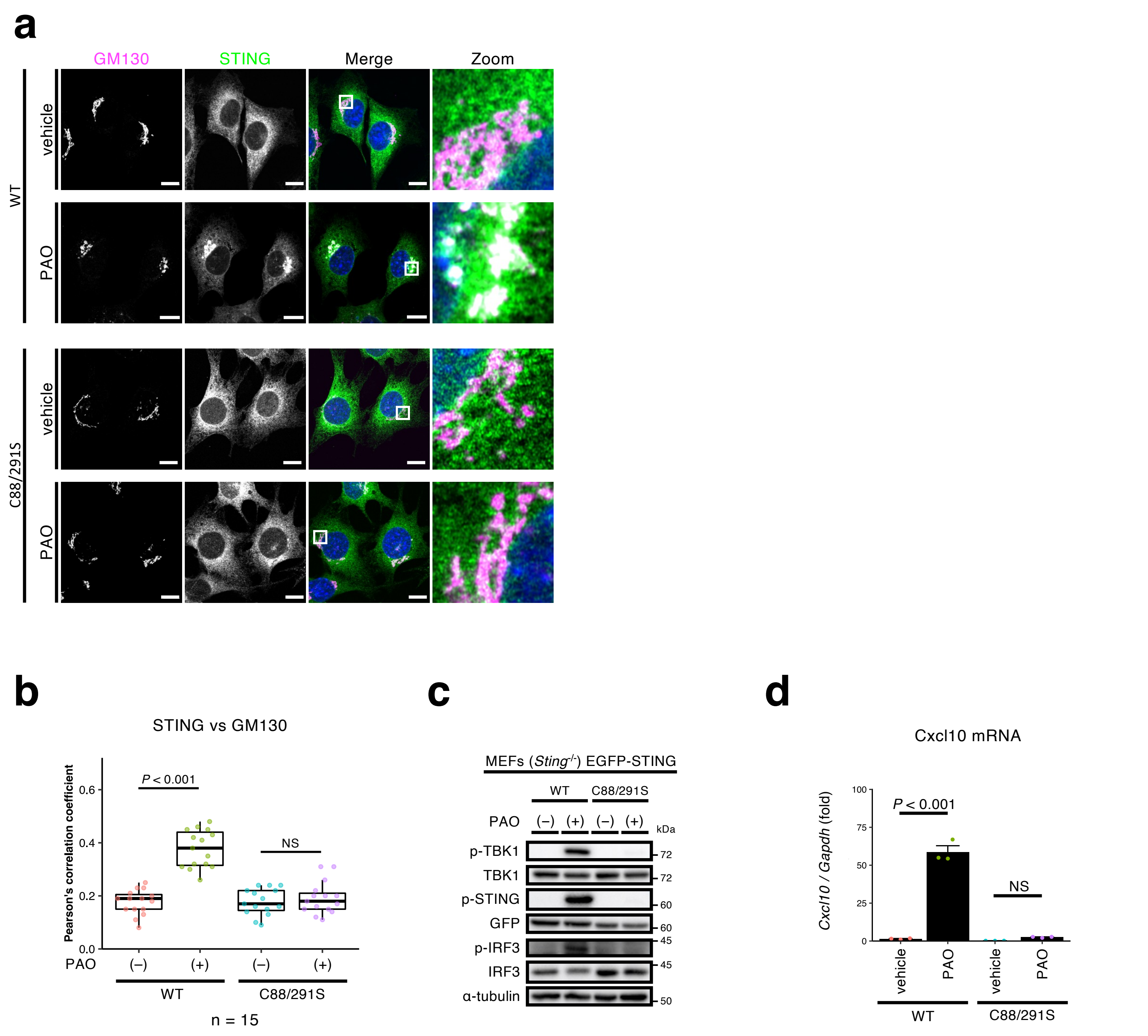

Supplementary Fig. 4 | STING C88/291S variant did not respond to PAO
a *Sting*$^{-/-}$ MEFs expressing EGFP-STING (C88S/C291S) were treated with PAO (1 μM) for 1 h. Cells were fixed, permeabilized, and stained for GM130 (a Golgi protein, magenta). Nuclei were stained with DAPI (blue). Scale bars, 10 μm. **b** The Pearson's correlation coefficient between EGFP-STING and GM130 in (a) is shown. Data are presented in box-and whisker plots (n = 15). **c** *Sting*$^{-/-}$ MEFs expressing EGFP-STING (C88S/C291S) were treated with PAO (1 μM) for 1 h. Cell lysates were then prepared and analyzed by western blot. **d** Cells were treated with PAO for 1 h followed by 4 h incubation without PAO. The expression of Cxcl10 was quantified by qRT-PCR.

Supplementary Fig. 5

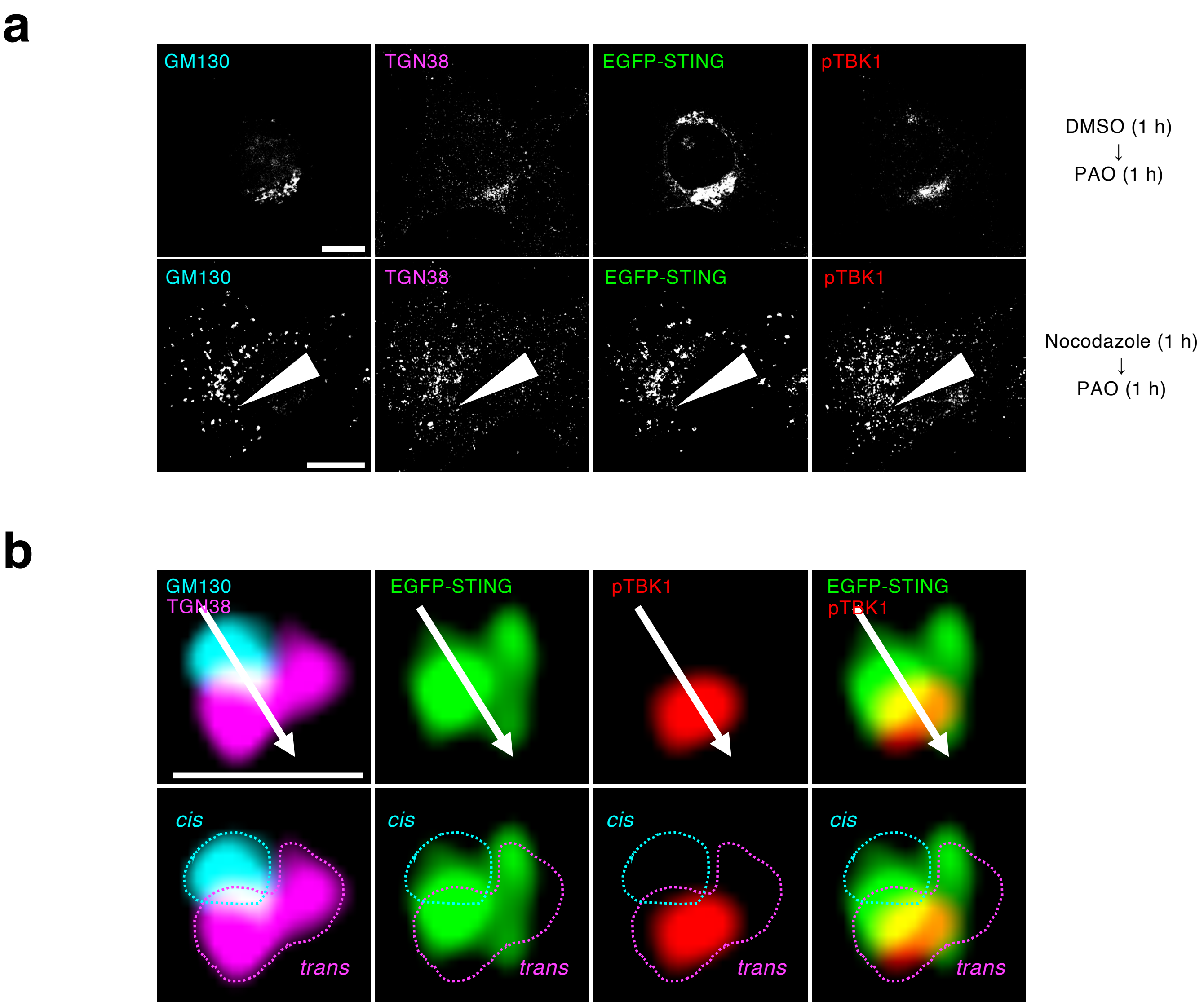

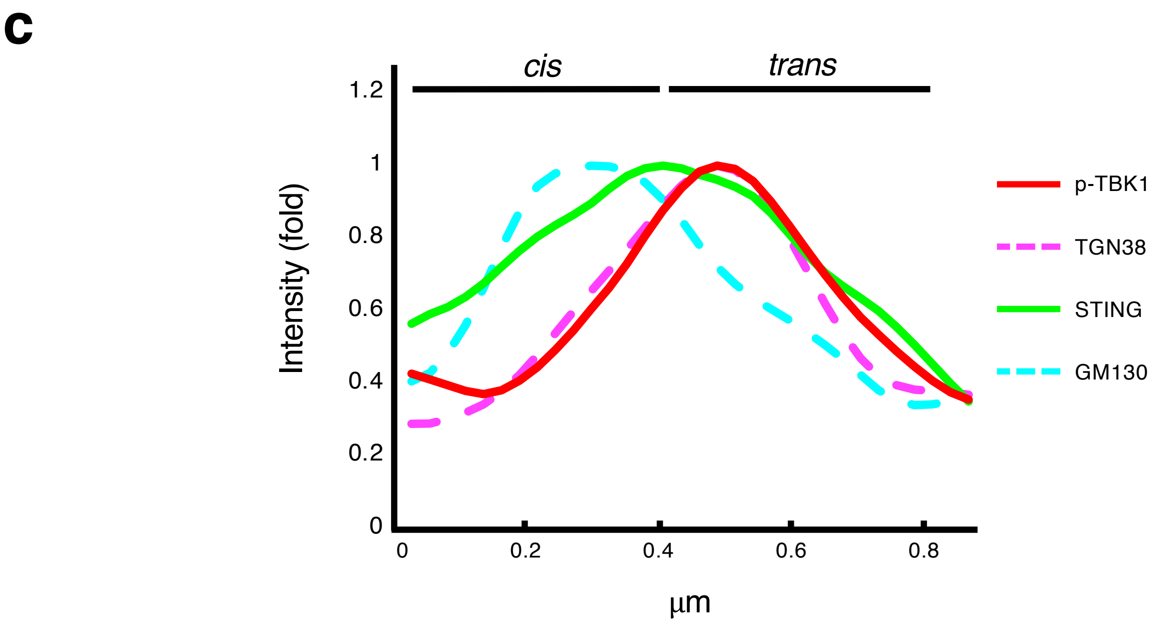

Supplementary Fig. 5 (continued)

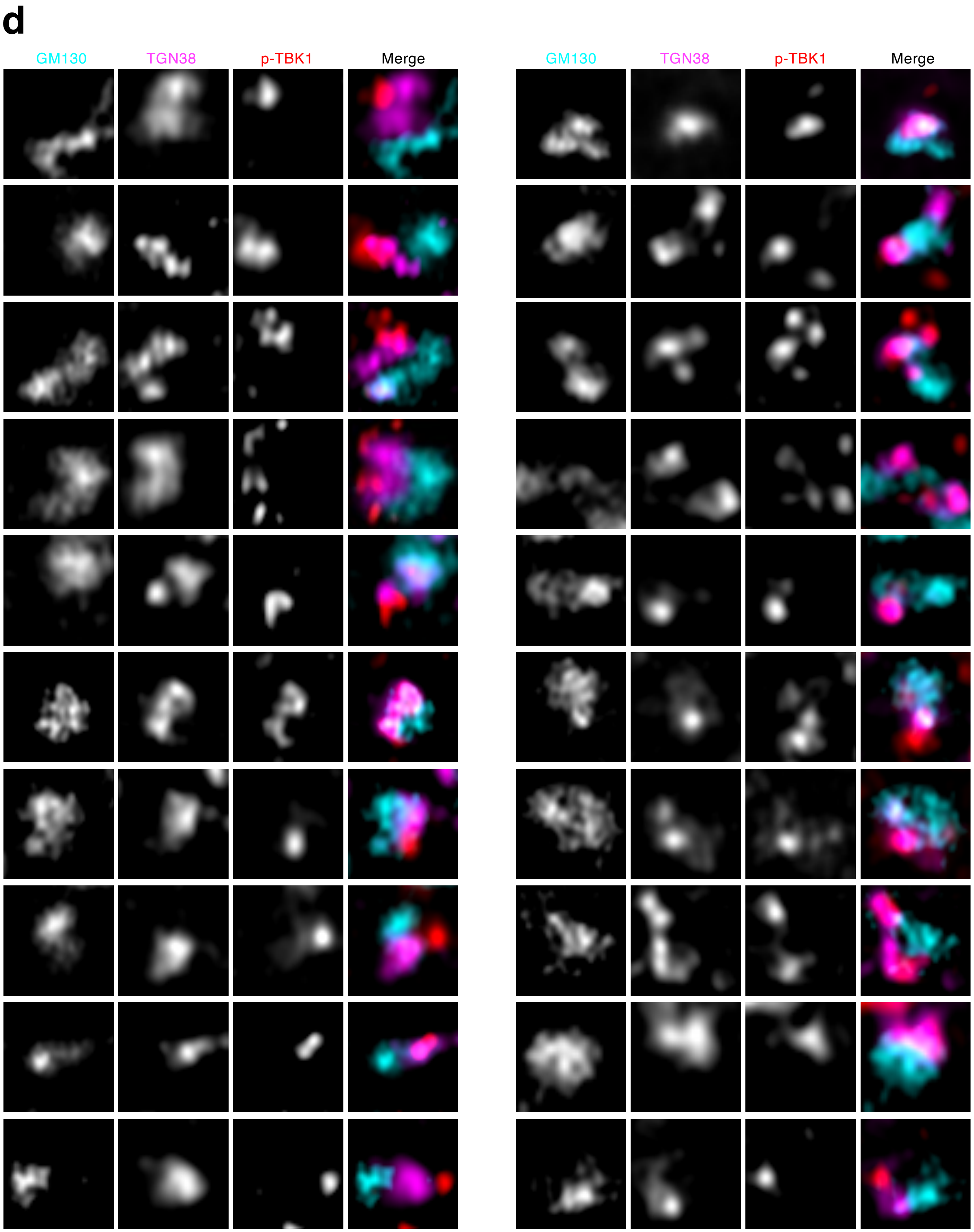

Supplementary Fig. 5 | STING activation with PAO resulted in the phosphorylation of TBK1 at the TGN
a EGFP-STING expressing *Sting*$^{-/-}$ MEFs were treated with nocodazole (2.5 μM) for 1 h, followed by stimulation with PAO (1 μM) for 1 h. Cells were fixed, permeabilized, and stained for phosphorylated TBK1 (red), GM130 (a CGN protein, cyan) and TGN38 (a TGN protein, magenta). Scale bars, 10 μm. **b** One mini-Golgi indicated by the arrowhead in (**a**) was magnified. The cis- and trans-regions of the mini-Golgi were outlined in the images at the bottom row. Scale bar, 1 μm. **c** Fluorescence intensity profile along the arrows in (**b**) is shown. **d** Magnified images of 20 mini-Golgi stacks from 5 cells are shown.

Supplementary Fig. 6

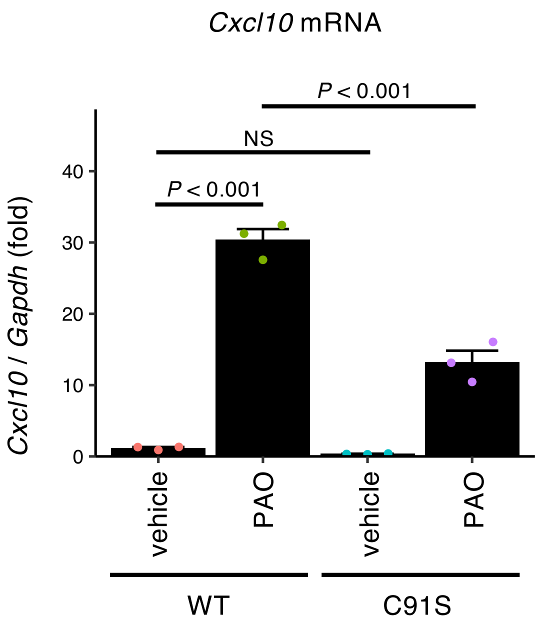

Supplementary Fig. 6 | STING C91S had the reduced activity to induce the expression of *Cxcl10*
Sting-/- MEFs expressing EGFP-mouse STING (C91S) were treated with PAO (1 µM) for 1 h, followed by 3 h incubation without PAO. The expression of *Cxcl10* was quantified by qRT-PCR. Data are mean ± s.e.m. from three independent experiments.

Supplementary Fig. 7

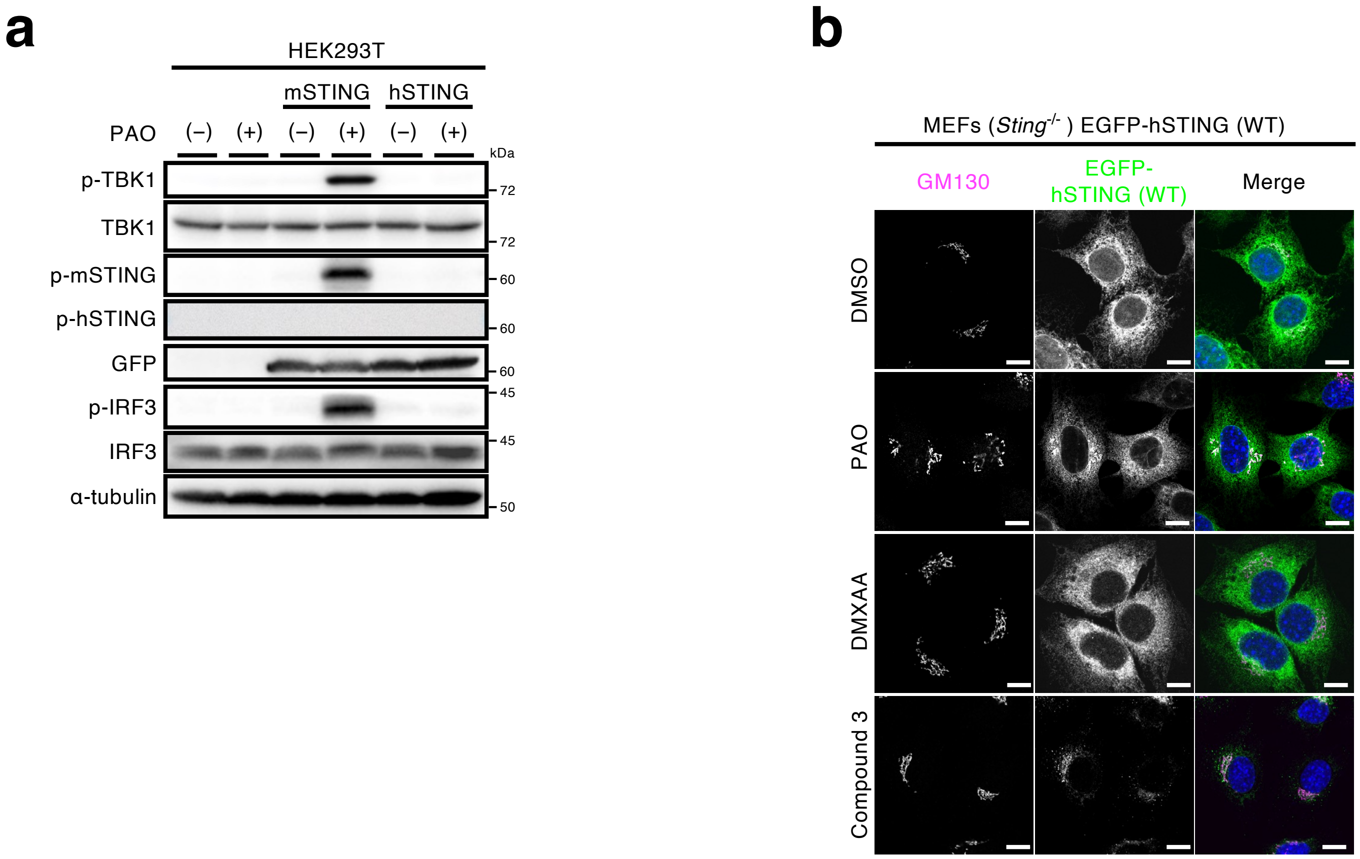

Supplementary Fig. 7 | PAO could not activate human STING

a EGFP-mouse STING or EGFP-human STING was stably expressed in HEK293T cells. Cells were treated with PAO (1 μM) for 1 h. Cell lysates were then prepared and analysed by western blot. **b** EGFP-human STING was stably expressed in *Sting*$^{-/-}$ MEFs. Cells were treated with vehicle, PAO (1 μM), mouse STING-specific agonist DMXAA (25 μg ml^{-1}), or human STING agonist Compound 3 (1 μM) for 1 h. Cells were fixed, permeabilized, and stained for GM130 (a Golgi protein, magenta). Nuclei were stained with DAPI (blue). No translocation of human STING from the ER was observed with PAO. Scale bars, 10 μm.
